# *Coxiella burnetii* in 3 Species of Turtles in the Upper Midwest, United States

**DOI:** 10.3201/eid2712.211278

**Published:** 2021-12

**Authors:** William E. Sander, Richard King, William Graser, Joshua M. Kapfer, Aubrey I. Engel, Laura Adamovicz, Matthew C. Allender

**Affiliations:** University of Illinois Urbana‒Champaign, Urbana, Illinois, USA (W. E. Sander, A.I. Engel, L. Adamovicz, M.C. Allender);; Northern Illinois University, DeKalb, Illinois, USA (R. King);; Kane County Forest Preserve, Geneva, Illinois, USA (W. Graser);; University of Wisconsin‒Whitewater, Whitewater, Wisconsin, USA (J.M. Kapfer)

**Keywords:** Coxiella burnetii, bacteria, Q fever, reptiles, turtles, sentinel species, reportable disease, zoonoses, Upper Midwest, Illinois, Wisconsin, United States

## Abstract

*Coxiella burnetii*, the causative bacterium of the zoonotic disease Q fever, has been documented in many different species. We describe documented turtles that were PCR positive for *C. burnetii* from multiple locations in Illinois and Wisconsin, USA. Assessing the conservation implications, reservoir potential, and zoonotic risk requires further research.

Two studies have identified *Coxiella burnetii* in poikilotherms (vertebrates that cannot regulate body temperature physiologically); both studies originated in India. Two tortoises had antibodies to *C. burnetii* by capillary agglutination testing of their serum samples in Uttar Pradesh (*1*). Additional reptiles, including snakes and skinks, had serum samples positive for *C. burnetii* in a separate study in Karnataka (*2*). Although both studies are useful in clarifying how this bacterium might interface with reptiles, there is no other evidence to support the role played by this large class of vertebrates (*3*). Furthermore, serologic assays applied to species that they were not designed for are difficult to interpret (Appendix).

Serologic testing, typically using indirect immunofluorescence assay, is the primary method used to diagnose *C. burnetii* infection, which causes Q fever in humans and coxiellosis in domestic ruminants (*4*). Additional serologic testing includes complement fixation and ELISA (*5*). Serologic assay benefits include commercial availability and insights into acute, treated, and chronic patients, depending on titers (*6*). Several PCR-based assays have been developed for detection of *C. burnetii* in samples from nontraditional mammals, birds, and arthropods (*7*). PCR provides a simple and reliable method for detection of the bacterium even retrospectively from tissues (*6*). Therefore, we tested turtles from multiple locations in Illinois and Wisconsin, USA, for *C. burnetii*.

This study was approved by the institutional animal care and use committees of the University of Illinois (20258), Northern Illinois University (LA16–0016), and University of Wisconsin‒Whitewater (K145011020Q). The Wildlife Epidemiology Laboratory, based at the University of Illinois College of Veterinary Medicine, continually conducts long-term, prospective health assessments of several turtle species across Illinois and neighboring states in natural habitats. Reptiles can be an excellent proxy for the health of environments, and many turtle species have small home ranges with diverse diets reflecting local conditions (*8*).

As part of these annual surveys, turtle species collected have various morphometric data, blood samples, or oral and cloacal swab specimens obtained before being released. Several diagnostic tests are performed with these samples, such as PCR screening for several pathogens, including *C. burnetii*. Other pathogenic organisms include *Ambystoma tigrinum* virus, Bohle iridovirus, Terrapene herpesvirus 1, Terrapene herpesvirus 2, epizootic hematopoietic necrosis virus, *Emydomyces testavorans*, frog virus 3, Emydid herpesvirus 1, Emydoidea herpesvirus 1 (in Blanding’s turtles), *Mycoplasma agassizii*, *M. testudineum*, *Salmonella* spp., and Testudinid herpesvirus 2 (*9*).

We extracted DNA from frozen, combined oral/cloacal swab specimens from each turtle by using the DNA Blood Mini Kit (QIAGEN, https://www.qiagen.com). We assessed spectrophotometrically DNA concentration and purity by using NanoDrop 1000 (Thermo Fisher Scientific Inc., https://www.thermofisher.com). We performed quantitative PCR by using a QuantStudio3 Real Time PCR System (Applied Biosystems, https://www.thermofisher.com) and a TaqMan primer‒probe assay targeting the *C. burnetti icd* gene as described (*10*). 

We assayed all samples, standards, and nontemplate controls in triplicate and quantified positive samples by using a 7-point standard curve (10^1^–10^7^ target copies). Samples were considered positive if all 3 replicates had a lower cycle threshold value than the lowest detected standard dilution. We used a highly sensitive and specific quantitative PCR for *C. burnetti.*

During 2019, samples from 5/605 turtles encountered across 8 counties showed positive results for quantitative PCRs, indicating presence of *C. burnetii* (Figure). We collected positive samples from 3 Blanding’s turtles (*Emydoidea blandingii*), 1 painted turtle (*Chrysemys picta*), and 1 ornate box turtle (*Terrapene ornata*). These positive turtles were found in Kane and Lee Counties in Illinois and Sauk County in Wisconsin. We did not perform serologic analysis for these animals. One Blanding’s turtle had a microchip and transmitter, was sampled again during 2020, and showed a negative PCR result. All of these turtles were found within a 1-hour drive to the Illinois‒Wisconsin state border within protected preserves. However, the 3 locations in which the 5 turtles varied in proximity to farms, livestock, industry, residential areas, and major highways; we found no geographic associations. All other screening tests showed negative results for pathogenic organisms for these 5 animals.

**Figure:**
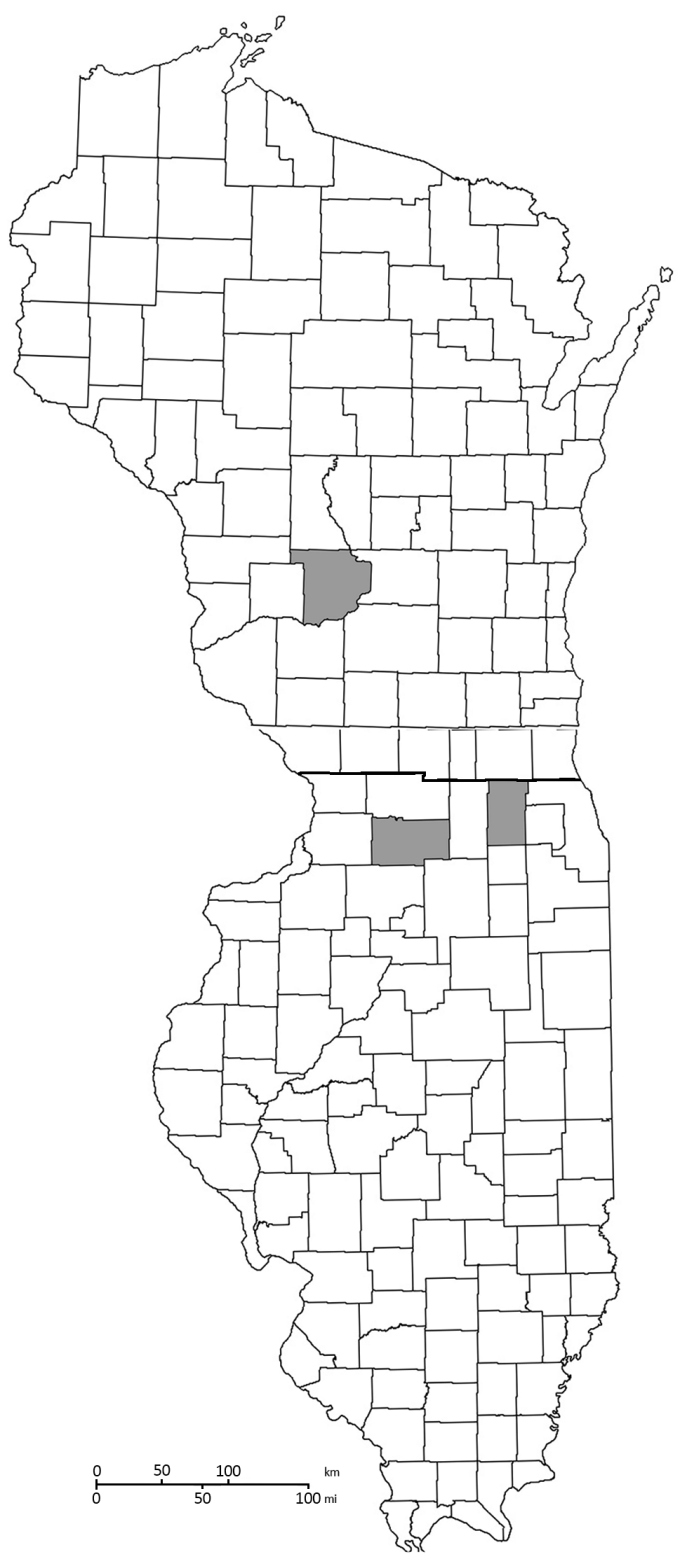
Location (gray areas) of turtles PCR positive for *Coxiella burnetii*, by county, Wisconsin (top) and Illinois (bottom), USA.

*C. burnetii* is a ubiquitous bacterium that has been found in many different species, often without pathogenicity (*4*). A variety of species of turtles are sampled annually in Illinois and surrounding areas through the Wildlife Epidemiology Laboratory. Over time, the testing for various organisms has expanded, especially as additional tests are validated. Screening for the bacterium that causes Q fever has been conducted for many species but infrequently in poikilotherms. These results show that the bacteria can be detected in these species and should be further researched to understand additional sources of this reportable disease, including potential management or regulatory decisions.

Continued investigation and screening in poikilotherms for zoonotic pathogens should be prioritized to understand the potential risk from additional hosts. The pet trade is a potential avenue of risk for exposure between humans and turtles. As these pathogens of concern are better characterized, the implications of different and varied hosts will drive the need for continued One Health research and dialogue between environmental, animal, and human health professionals.

Appendix Additional information on *Coxiella burnetii* in 3 species of turtles in the Upper Midwest, United States.
