# Supplementary material for: Coxiella burnetii in 3 Species of Turtles in the Upper Midwest, United States
Source: Emerg Infect Dis. 2021 Dec;27(12):3199–202. doi: 10.3201/eid2712.211278 (PMC8632166; doi:10.3201/eid2712.211278)
Supplement: Appendix — Additional information on Coxiella burnetii in 3 species of turtles in the Upper Midwest, United States. [file 21-1278-Techapp-s1.pdf]

Article DOI: https://10.3201/eid/2712.211278

Coxiella burnetii in 3 Species of Turtles in the Upper Midwest, United States

Appendix

Q fever has been a nationally reportable disease in the United States since 1999 (*1*). Since then, cases continue to increase every year, and there is an estimated overall seroprevalence of 3.1% (*1*). Surveillance and reporting of Q fever are key components of public health education and disease prevention efforts (*1*). As a zoonotic disease, persons at particular risk for Q fever include farmers, veterinarians, and abattoir workers because they have the highest level of contact with goats, sheep, and cows. However, additional case reports have emerged for persons who do not have these occupations. This fact is particularly concerning for persons who have meningoencephalitis or myocarditis, in whom Q fever can be severe and not self-limiting, as it is in many persons (*2*). For most persons, the disease does not show symptoms, but some persons might have fever, pneumonia, or hepatitis (*2*).

The main sources for *Coxiella burnetii* in persons are domestic ruminants, including goats, sheep, and cows; the highest shedding occurs in birth products, followed by urine, feces, and milk (*3*). Infected animals might not show or show clinical signs, including infertility, stillbirth, abortion, and mastitis (*4*). There are many identified reservoirs for *C. burnetii*, including many species of mammals, birds, and arthropods (*4*). Wildlife can constitute a reservoir with documented cases from kangaroo and wallabies in Australia (*5*) and the 3-toed sloth in French Guiana (*6*). Because *C. burnetii* has been isolated from many arthropods, including 40 hard tick species, 14 soft tick species, bed bugs, flies, and mites, these arthropods might play a role in transmission of the infectious agent (*7*).

References

1. Anderson A, Bijlmer H, Fournier P-E, Graves S, Hartzell J, Kersh GJ, et al. Diagnosis and management of Q fever—United States, 2013: recommendations from CDC and the Q Fever Working Group. MMWR Recomm Rep. 2013;62:1–30. PubMed

2. Maurin M, Raoult D. Q fever. Clin Microbiol Rev. 1999;12:518–53. PubMed https://doi.org/10.1128/CMR.12.4.518

3. Rodolakis A. Q Fever in dairy animals. Ann N Y Acad Sci. 2009;1166:90–3. PubMed https://doi.org/10.1111/j.1749-6632.2009.04511.x

4. Tissot-Dupont H, Raoult D. Q fever. Infect Dis Clin North Am. 2008;22:505–14, ix. PubMed https://doi.org/10.1016/j.idc.2008.03.002

5. Stevenson S, Gowardman J, Tozer S, Woods M. Life-threatening Q fever infection following exposure to kangaroos and wallabies. BMJ Case Rep. 2015;2015:3. PubMed https://doi.org/10.1136/bcr-2015-210808

6. Davoust B, Marié J-L, Pommier de Santi V, Berenger J-M, Edouard S, Raoult D. Three-toed sloth as putative reservoir of *Coxiella burnetii*, Cayenne, French Guiana. Emerg Infect Dis. 2014;20:1760–1. PubMed https://doi.org/10.3201/eid2010.140694

7. Eldin C, Mélenotte C, Mediannikov O, Ghigo E, Million M, Edouard S, et al. From Q fever to *Coxiella burnetii* infection: a paradigm change. Clin Microbiol Rev. 2017;30:115–90. PubMed https://doi.org/10.1128/CMR.00045-16
